# Combined analysis of serum γ-glutamyl transferase isoenzyme II, α-L-fucosidase and α-fetoprotein detected using a commercial kit in the diagnosis of hepatocellular carcinoma

**DOI:** 10.3892/etm.2012.783

**Published:** 2012-10-31

**Authors:** JING ZHU, FENG JIANG, HONG-BING NI, MING-BING XIAO, BU-YOU CHEN, WEN-KAI NI, CUI-HUA LU, RUN-ZHOU NI

**Affiliations:** 1 Departments of Gastroenterology; 2 Medical Laboratory Center and; 3 Radiochemotherapy, Affiliated Hospital of Nantong University, Nantong, Jiangsu 226001, P.R. China

**Keywords:** γ-glutamyl transferase isoenzyme II, α-L-fucosidase, α-fetoprotein, hepatocellular carcinoma, diagnosis

## Abstract

γ-glutamyl transferase isoenzyme II (GGT-II) is a sensitive biomarker of hepatocellular carcinoma (HCC). However, numerous disadvantages of the traditional manual method affected its application. The commercial kit provided a convenient and fast method for the determination of GGT-II levels. The purposes of the present study were to compare the reproducibility and sensitivity between the manual and commercial kit methods and to evaluate the diagnostic efficiency for HCC with the combined analysis of GGT-II, α-L-fucosidase (AFU) and α-fetoprotein (AFP). In patients with various liver diseases (HCC, liver cirrhosis and chronic hepatitis) and normal subjects, GGT-II was detected by manual and commercial polyacrylamide gel electrophoresis (PAGE). The levels of AFU and AFP were assayed by colorimetry and a chemiluminescence immunoassay, respectively. The commercial PAGE had equal diagnostic efficiency with traditional manual PAGE and no significant differences were observed in intra- and average-gel reproducibility and GGT-II sensitivities between the manual and commercial PAGE (P>0.05). The incidence of GGT-II detected by commercial PAGE in HCC patients was 84.1% and <8% in benign liver disease. The levels of AFU and AFP in the benign liver diseases and normal subjects were lower than those in HCC. According to the cut-off value obtained by receiver operating characteristic curves, a total of 56.6 and 59.3% of HCC patients (64 out of 113 and 67 out of 113) had AFU >636.5 *μ*mol/l h and AFP >44.0 *μ*g/l, respectively. There were no significant correlations between GGT-II and AFU or AFP. Combined detection of GGT-II with AFU or AFP increased the diagnostic sensitivity to 92.9 and 93.8%, respectively. These results suggest that commercial PAGE provides a simple and reproducible method for GGT-II detection. Combined determination of GGT-II with AFU or AFP exhibited superior sensitivity and specificity for the diagnosis of HCC.

## Introduction

Hepatocellular carcinoma (HCC) is the second and the fifth most frequent type of cancer in China and the world, respectively. The five-year survival rate is <12% and mortality remains equal with morbidity (1,2). The occurrence and development of HCC is a complex multifactor and multistep process, mainly associated with chronic and persistent infection with the hepatitis virus, particularly the hepatitis B virus (HBV) and hepatitis C virus (HCV) (3,4).

HCC is highly malignant and metastasizes readily at the early stages and its prognosis depends mainly on early diagnosis. Imaging (BUS, CT, MRI) and the detection of serum tumor markers are fundamental methods of identification in asymptomatic patients with HCC. However, at present no single diagnostic method is able to meet the sensitivity and specificity criteria required. In terms of serum markers, α-fetoprotein (AFP) is the preferred serum marker for the diagnosis and monitoring of HCC but it is negative in ∼40% patients with early stage HCC. Even in advanced HCC, the concentrations of AFP may be normal in 15–30% of patients (5). Additional HCC markers, including γ-glutamyl transferase isoenzyme II (GGT-II), α-L-fucosidase (AFU), Golgi protein-73 (GP73) and transforming growth factors α and β (TGF-α and TGF-β), have been used to elevate the diagnostic sensitivity, specificity and early detection levels. However, there is no absolute positive and universal diagnostic marker for HCC. Therefore, it is suggested that markers be simultaneously evaluated in order to enhance the detection of HCC (6–8).

γ-glutamyl transpeptidase (GGT; EC 2.3.2.2) is a membrane-bound enzyme which hydrolyzes γ-glutamyl or transfers it to a suitable acceptor to degrade glutathione and its conjugates. Serum GGT has been used in the diagnosis of liver diseases, as it exhibits tissue-specific expression. In damaged hepatocytes, particularly in hepatocarcinogenesis, since GGT is released into the blood from hepatic tissues, significant changes occur in serum GGT activity (9). However the total activity of GGT has a significant overlap with various liver diseases which limits its value in diagnosis. The identification of hepatoma-specific GGT (GGT-II) provides an efficacious serum marker for HCC which is regarded as an early enzyme marker of precancerous and cancerous processes. In the serum of HCC patients, GGT-II as a heterodimeric glycoprotein has different electrophoretic mobility and may be separated from normal GGT isoenzymes by polyacrylamide gel electrophoresis (PAGE) (10–12). However, more disadvantages affect traditional manual PAGE and prevent its wider application. It has been difficult to maintain uniform intra- and inter-laboratory reproducibility since all reagents of the experiment are artificially prepared in each laboratory. Moreover, care must be taken when preparing the gel since acrylamide is a strong neurotoxin in its powdered and liquid forms. Commercial PAGE kits have solved these problems and shortened the electrophoretic run time to only 4 h. The main objective of the present study was to compare reproducibility and sensitivity between manual and commercial PAGE for the detection of GGT-II and evaluate the diagnostic efficiency of a combined assay of GGT-II, AFU and AFP.

## Patients and methods

### Patients and specimens

The serum specimens of the HCC group were obtained from 113 patients (median age, 55.3 years; 95 males and 18 females) from the Affiliated Hospital of Nantong University (Nantong, China). Of the patients, 85.0% (96/113) were hepatitis B surface antigen (HBsAg) carriers, 9.7% (11/113) were HCV antibody-positive, 2.7% (3/113) had alcoholic hepatitis and 2.7% (3/113) had autoimmune hepatitis. The other study groups included liver cirrhosis (LC; 51 patients; 33 males, 18 females; median age, 53 years), chronic viral hepatitis (CH; 21 patients; 16 males, 5 females; median age, 44 years) and normal control subjects group (NC; 14 males, 14 females; median age, 25 years). All studied cases were diagnosed by hepatitis markers, blood biochemical tests, imaging or pathohistological examination. No patients received any therapy before the blood specimens were collected.

### GGT-II detected by manual PAGE

The separation of GGT-II was performed by manual PAGE on a vertical slab apparatus (12,13). In short, polyacrylamide staging gel containing three layers of separation gel (7.7, 11.5 and 15.4%) and one layer of concentration gel (4.2%) on top was loaded into a vertical slab electrophoresis bath (Model JSZH-I, Jiangsu Zongheng Co., Ltd., Nantong, China). After infusion with negative and positive buffer solution, 20 *μ*l of serum was mixed with 20 *μ*l of 40% sucrose bromophenol blue solution and introduced into the sample holes of the concentration gel, then electrophoresed at a constant voltage of 60 V for 4 h, then 100 V for 12 h. Subsequently, the polyacrylamide gel removed from the electrophoresis bath was covered over with a cellulose-acetate sheet soaked with GGT substrate solution (containing 24 mg γ-L-glutamyl-P-nitroanilide monohydrate, 100 *μ*l of 20% Tween-20, 100 *μ*l of 5% polyvinyl pyrrolidone, 5.0 ml of 0.1 mol/l tromethamine-glycylglycine buffer and 100 *μ*l of 10% NaNO_2_) and incubated at 37°C for 60 min. The cellulose-acetate sheet was then soaked with 5 ml staining solution (containing 10% trichloroacetic acid and 25% glycerol) and within ∼2 min the red bands of GGT isoenzyme appeared on the cellulose acetate sheet.

### GGT-II detected by commercial PAGE kit

The separation technique of the commercial kit (Jiangsu Zongheng Co., Ltd.) for GGT-II was also PAGE and was performed according to the manufacturer’s instructions.

### Reproducibility studies of manual PAGE and commercial PAGE for GGT-II

To determine the reproducibility of the manual PAGE and commercial PAGE for GGT-II, positive and negative controls of GGT-II (Jiangsu Zongheng Co., Ltd.) were electrophoresed nine times on the same and different gels from the manually prepared or commercial gels to determine intra- and average-gel reproducibility.

### Serum AFU concentration

The serum AFU activities were assayed colorimetrically using a semi-automatic biochemistry analyzer (BA-88). The cut-off value of serum AFU for HCC was obtained using a receiver operating characteristic (ROC) curve.

### Serum AFP concentration

According to the manufacturer’s instructions, the chemiluminescence immunoassay (Abbott, Chicago, IL, USA) was used to detected the serum levels of AFP.

### Statistical analysis

The Chi-square or Fisher’s exact test were used for any 2x2 tables. Serum AFU concentrations are presented as the mean ± SD and analyzed with F-tests. Serum AFP concentrations in patients with liver disease are presented as median (range) and analyzed with the rank sum test with regard to the skewness of the distribution of AFP concentrations. The diagnostic values of serum AFU and AFP were evaluated using an ROC curve. Spearman’s rank correlation was used to test the correlations between serum GGT-II, AFU and AFP. P<0.05 was considered to indicate statistically significant differences in all analyses. All statistical analysis was performed using the SPSS 17.0 software package (SPSS, Inc., Chicago, IL, USA).

## Results

### Reproducibility comparison between manual PAGE and commercial PAGE

Positive and negative controls of GGT-II were repeatedly electrophoresed on the same and different gels from the manually prepared or commercial gels. The results showed (Table I) that although the average reproducibility of the commercial PAGE (100%) was higher than that of the manual PAGE (94.4%), the intra- and average-gel reproducibility differences between the manual and commercial PAGE (P>0.05) were not statistically significant.

### Serum GGT-II determined by manual and commercial PAGE

**Figure 1:**
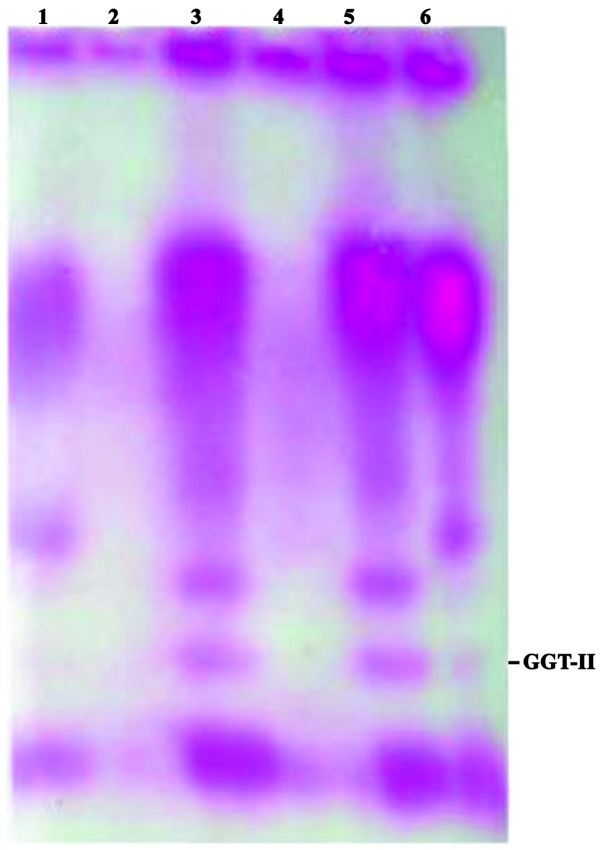
GGT-II-positive staining was shown in hepatocellular carcinoma. (1) Liver cirrhosis; (2) healthy control; (3, 5 and 6) hepatocellular carcinoma; (4) chronic hepatitis. GGT-II, γ-glutamyl transferase isoenzyme II.

Serum GGT-II was determined by manual and commercial PAGE in all cases. Several bands (up to 9) of the serum GGT isoenzymes were separated and labeled GGT-I, GGT-II and GGT III-IX, from the positive to negative pole according to the decreasing order of their electrophoretic mobility (Fig. 1). The diagnostic values of GGT-II in the sera of patients with malignant and benign liver diseases are shown in Table II. By manual and commercial PAGE, there were few false-positives of GGT-II among the patients with benign liver diseases and none among the normal subjects. The positive GGT-II was predominantly exhibited by HCC patients with sensitivities of 74.3 and 84.1% by manual and commercial PAGE, respectively, which were significantly higher than in the benign liver diseases and healthy subjects (P<0.05). However, no significant differences (P>0.05) were observed between manual and commercial PAGE.

### Serum levels of AFU and AFP

**Figure 2:**
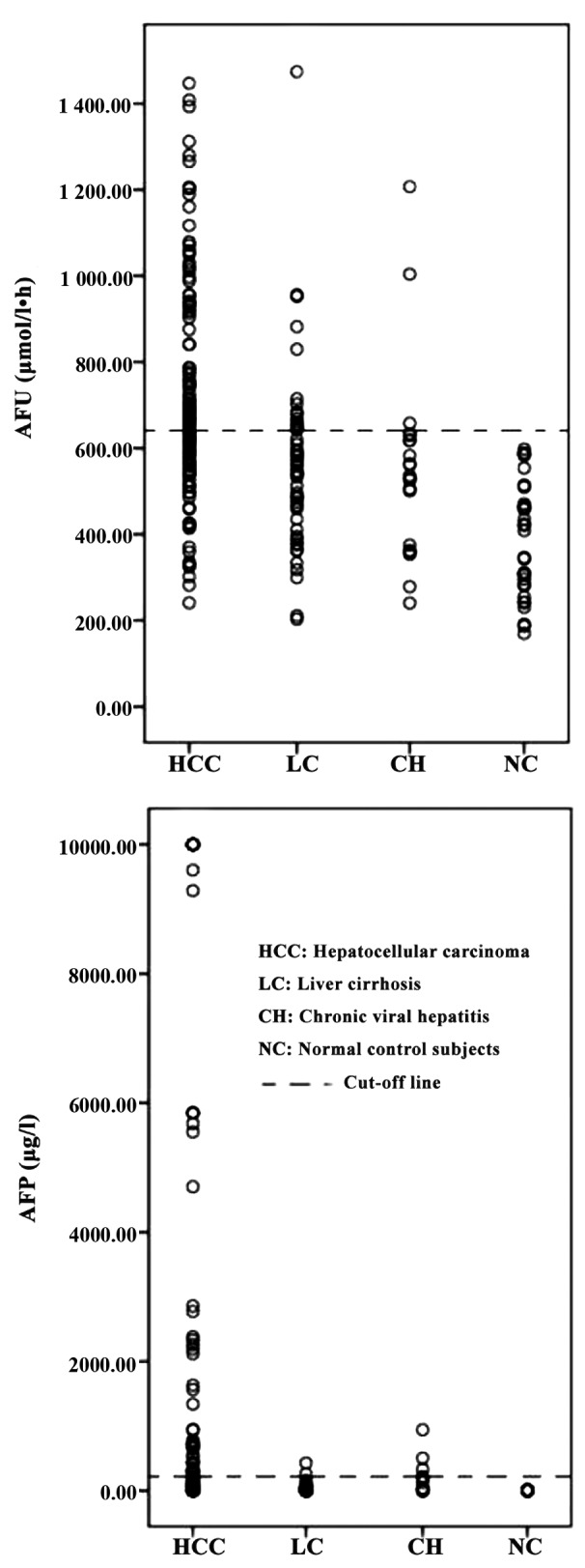
Scatterplot of serum levels of AFU and AFP in various liver diseases and normal control subjects. AFU, α-L-fucosidase; AFP, α-fetoprotein.

The results demonstrated that the levels of AFU (726.4±258.4 *μ*mol/l h) and AFP [321.1 (0.7–10000.0) *μ*g/l] were higher in the HCC group than in the other groups (Fig. 2, Table III, P<0.05).

**Figure 3:**
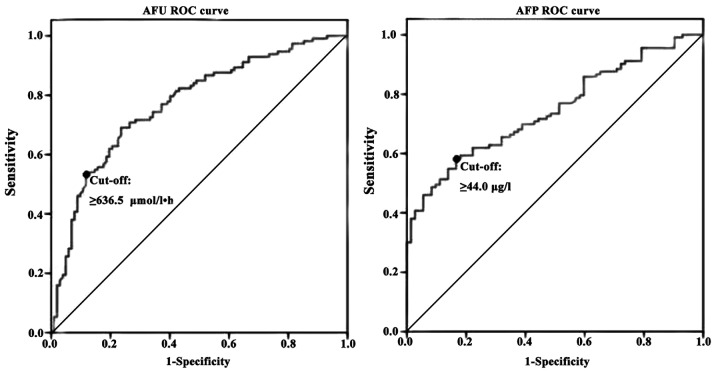
ROC curve of serum levels of AFU and AFP for the diagnosis of hepatocellular carcinoma. ROC, receiver operating characteristic; AFU, α-L-fucosidase; AFP, α-fetoprotein.

The cut-off values for optimal diagnostic efficiency determined by ROC curve analysis (Fig. 3) were 636.5 *μ*mol/l h for AFU and 44.0 *μ*g/l for AFP and the areas under the ROC curves were 0.772 (95% CI, 0.709–0.834; P<0.05) and 0.746 (95% CI, 0.677–0.814; P<0.05), respectively. According to the cut-off values, the sensitivities of AFU and AFP for the diagnosis of HCC were 56.6 and 59.3%, respectively (Table III).

### Serum positive GGT-II is complementary to the serum levels of AFU and AFP

Positive GGT-II was observed in 95 of 113 patients with HCC (84.1%) and among the 18 patients with negative GGT-II, 10 had elevated concentrations of AFU (≥636.5 *μ*mol/l h). No significant correlation was observed between GGT-II and the serum levels of AFU in HCC patients (r=0.142, P=0.383). Of 18 HCC patients with negative GGT-II, 11 had an AFP concentration above 44.0 *μ*g/l. Also, no significant correlation was observed between GGT-II and the serum levels of AFP (r=0.102, P=0.517).

The diagnostic sensitivity was significantly increased by combining the information on GGT-II, AFU and AFP levels (Table IV). The combination of GGT-II with AFU (a+b), GGT-II with AFP (a+c) and the three markers (a+b+c) had superior diagnostic sensitivities (92.9, 93.8 and 97.3%) to that (83.2%) from the combination of AFU with AFP (b+c) [P<0.05, (b+c) vs. (a+b), (a+c) and (a+b+c)]. However, the combination of three markers had the lowest diagnostic specificity (64.7%), which was significantly different from the combinations of AFU (78.4%) and GGT-II with AFP (77.5%) [P<0.05, (a+b) and (a+c) vs. (a+b+c)]. The combinations of GGT-II with AFU and GGT-II with AFP had the optimal diagnostic efficiency.

## Discussion

The level of AFP has been widely established as a classic HCC marker, but an elevated AFP level is also observed in certain benign liver diseases and other malignancies. Furthermore, false-negative or -positive results often occur for a number of reasons, including geographical and ethnic variations and different techniques being employed. For these reasons, it is necessary to perform a combined detection of various markers for the diagnosis of HCC. As mentioned previously, GGT-II and AFU are frequently used.

As an important and extensively distributed converting enzyme closely correlated with nucleic acid metabolism and biotransformation, changes of GGT sensitively reflect hepatocyte parenchymatous lesions (14). Previously, GGT has been widely studied in tumorigenesis, such as the dysfunction of DNA synthesis and nucleic acid metabolism. Certain researchers have observed a highly positive correlation between liver RNA level and hepatic GGT gene expression in a hepatoma model (15). The abnormal regulation of genes may be the result of the inactivation of tumor suppressor genes or the activation of proto-oncogenes initiated by carcinogens.

The separation of serum GGT isoenzymes using PAGE works mainly on the basis of their different electrophoretic mobilities. A number of researchers have reported different results for the hepatoma-specific GGT isoenzyme according to fractionation systems. Initially, the positive rate was only 27–63% (16–18). Xu *et al* subsequently reported that the GGT isoenzymes may be divided into 9 to 11 bands by vertical slab polyacrylamide gradient gel electrophoresis and a number of these bands (I, II and II bands, GGT-II) were observed to have a positive rate of 90% (13). In the present study, the positive rate of GGT-II in HCC was 74.3% using the traditional manual PAGE described by Xu *et al*, similar to the study by Cui *et al* (77.6%) (19). Previous studies suggest that GGT-II is important for the diagnosis of HCC, but certain disadvantages of the traditional manual method affected the sensitivity of GGT-II. Yao and Dong also demonstrated that GGT-II was a good marker, but there was no simple and easy detection method (20). Now, the use of commercial PAGE kits has solved this problem. In the present study, the reproducibility (100%) and diagnostic sensitivity (84.1%) of GGT-II detected by a commercial PAGE kit was higher than that of the traditional method. The commercial PAGE kit provided a simple, convenient method for clinical application.

As a glycosidase which is widespread in a variety of cell lysosomes in the human body, AFU is associated with acid hydrolysis of a variety of fucose-containing fucoglyco-conjugates. The activity of this liposomal enzyme is detectable and elevated activities are observed in the sera of HCC patients compared with chronic liver disease and healthy individuals (21). Early studies showed that the sensitivity and specificity of AFU for diagnosis of primary hepatocarcinoma were 70–80% (22,23). In contrast to AFP, the activity levels of AFU were not correlated with tumor magnitude and AFU was of value in the diagnosis of HCC patients with negative or low serum levels of AFP, particularly for small HCC (<5 cm) (24–26). However, elevated serum AFU is also detected in colorectal cancer, ovarian cancer and other malignancies. In addition, serum AFU levels change in diabetes, pancreatitis and hypothyroidism (27). In the present study, the sensitivity of AFU for HCC was only 56.6% which was lower than previous reports, owing to a higher cut-off value (636.5 *μ*mol/l h) with a higher diagnostic specificity (82.4%). To improve the diagnostic sensitivity, the combined detection of AFU with other tumor markers should be commonly used in clinical practice.

Spearman’s rank correlation analysis showed that positive GGT-II was not significantly correlated with either AFU or AFP, suggesting that these three markers are complementary in the diagnosis of HCC. The sensitivity of HCC detection using GGT-II was 84.1%. This sensitivity increased to 92.9 and 93.8% when combined with AFU or AFP, respectively. The combination of three markers yielded a 97.3% detection sensitivity but the lowest diagnostic specificity (64.7%). In summary, the combinations of GGT-II with AFU or AFP had optimal diagnostic efficiency. We propose that GGT-II be measured with AFU or AFP to improve the detection sensitivity of HCC.

## Figures and Tables

**Table I:** Reproducibility comparison between manual and commercial PAGE.

Method	Positive control reproducibility (positive/total, %)			Negative control reproducibility (negative/total, %)			Average reproducibility (%)
	Gel 1	Gel 2	Gel 3	Gel 1	Gel 2	Gel 3	
Manual PAGE^a^	7/9 (77.8)	9/9 (100)	8/9 (88.9)	9/9 (100)	9/9 (100)	9/9 (100)	51/54 (94.4)
Commercial PAGE	9/9 (100)	9/9 (100)	9/9 (100)	9/9 (100)	9/9 (100)	9/9 (100)	54/54 (100)

a P>0.05, all values of manual PAGE compared with those in commercial PAGE (Fisher’s exact test). PAGE, polyacrlyamide gel electrophoresis.

**Table II:** Comparison of diagnostic sensitivity of GGT-II detected by manual or commercial PAGE.

		GGT-II (manual PAGE)		GGT-II (commercial PAGE)	
Groups	n	n	%	n	%
HCC	113	84	74.3^a,b^	95	84.1^a^
LC	51	3	5.9^b^	4	7.8
CH	21	1	4.8^b^	1	4.8
NC	30	0	0.0^b^	0	0.0

a P<0.05, HCC group compared with the other groups;

b P>0.05, the sensitivities of GGT-II in all groups detected by manual PAGE were compared with those detected by commercial PAGE (Chi-square test or Fisher’s exact test). GGT-II, γ-glutamyl transferase isoenzyme II; HCC, hepatocellular carcinoma; LC, liver cirrhosis; CH, chronic viral hepatitis; NC, normal control; PAGE, polyacrylamide gel electrophoresis.

**Table III:** Serum levels of AFU and AFP in various liver diseases and normal control subjects.

		AFU (*μ*mol/l h)				AFP (*μ*g/l)			
Groups	n	Mean ± SD	F^a^	P-value^a^	≥636.5 (%)	Median (range)	Z^b^	P-value^b^	≥44.0 (%)
HCC	113	726.4±258.4			64 (56.6)^c^	321.1 (0.7–10000.0)			67 (59.3)^c^
LC	51	562.7±208.6	−163.71	0.001	15 (29.4)	7.3 (0.4–860.7)	4.723	0.000	14 (27.5)
CH	21	554.4±222.4	−172.01	0.021	3 (14.3)	14.2 (0.7–240.3)	4.839	0.000	5 (23.8)
NC	30	382.4±133.0	−344.05	0.000	0 (0.0)	2.3 (0.2–9.7)	5.898	0.000	0 (0.0)

a F and P-values were calculated by F-tests;

b Z and P-values were calculated by rank-sum tests;

c P<0.05 and calculated by Chi-square or Fisher’s exact tests, while the HCC group was compared with the other groups. AFU, α-L-fucosidase; AFP, α-fetoprotein; HCC, hepatocellular carcinoma; LC, liver cirrhosis; CH, chronic viral hepatitis; NC, normal control.

**Table IV:** Complementary value of GGT-II, AFU and AFP to diagnose hepatocellular carcinoma.

Markers	Sensitivity (%)	Specificity (%)	Accuracy (%)
Positive GGT-II (a)	84.1 (95/113)^a^	95.1 (97/102)^d^	89.3 (192/215)
Positive AFU (b)	56.9 (64/113)^b^	82.4 (84/102)^e^	68.8 (148/215)
Positive AFP (c)	59.3 (67/113)^b^	81.4 (83/102)^e^	69.8 (150/215)
a+b	92.9 (105/113)	78.4 (80/102)^f^	86.0 (185/215)
a+c	93.8 (106/113)	77.5 (79/102)^f^	86.0 (185/215)
b+c	83.2 (94/113)^c^	68.8 (70/102)	76.3 (164/215)
a+b+c	97.3 (110/113)	64.7 (66/102)	81.9 (176/215)

a P<0.05, a vs. b, c, (a+b), (a+c) and (a+b+c);

b P<0.05, b and c vs. (a+b), (a+c), (b+c) and (a+b+c);

c P<0.05, (b+c) vs. (a+b), (a+c) and (a+b+c);

d P<0.05, a vs. other groups;

e P<0.05, b and c vs. (b+c) and (a+b+c);

f P<0.05, (a+b) and (a+c) vs. (a+b+c). Statistical analyses were performed using Chi-square or Fisher’s exact tests. GGT-II, γ-glutamyl transferase isoenzyme II; AFU, α-L-fucosidase; AFP, α-fetoprotein.
